# Self-reported health problems in a health risk appraisal predict permanent work disability: a prospective cohort study of 22,023 employees from different sectors in Finland with up to 6-year follow-up

**DOI:** 10.1007/s00420-019-01497-2

**Published:** 2019-11-30

**Authors:** Minna Pihlajamäki, Jukka Uitti, Heikki Arola, Mikko Korhonen, Tapio Nummi, Simo Taimela

**Affiliations:** 1 Faculty of Medicine and Health Technology, Tampere University, Tampere, Finland; 2 Clinic of Occupational Medicine, Tampere University Hospital, Tampere, Finland; 3 Finnish Institute of Occupational Health, Tampere, Finland; 4 Terveystalo, Jaakonkatu 3b, 00100 Helsinki, Finland; 5 Faculty of Information Technology and Communication Sciences, Tampere University, Tampere, Finland; 6 Department of Orthopedics and Traumatology, University of Helsinki, Helsinki, Finland; 7 Evalua International, PO Box 35, 02661 Espoo, Finland

**Keywords:** Health risk appraisal, Work disability, Disability retirement, Cumulative incidence function

## Abstract

**Purpose:**

Work disability (WD) as a medico-legal concept refers to disability benefits (DB) that are granted due to diseases that permanently reduce work ability. We studied whether an occupational healthcare instrument for the prediction of sickness absence (SA) risk—a health risk appraisal (HRA)—also predicts permanent WD.

**Methods:**

HRA results were combined with registry data on DB of 22,023 employees from different industry sectors. We analysed how the HRA risk categories predict DB and considered occupational group, gender, age, and prior SA as confounding variables. Cumulative incidence function illustrates the difference between the HRA risk categories, and the Fine–Gray model estimates the predictors of WD during 6-year follow-up.

**Results:**

The most common primary reasons for permanent WD were musculoskeletal (39%) and mental disorders (21%). Self-reported health problems in the HRA, labelled as “WD risk factors”, predicted DB when controlling for age and prior SA. Hazard ratios were 10.9 or over with the lower limit of the 95% confidence interval 3.3 or over among those with two simultaneous WD risk factors. 14% of the females and 17% of the males with three or more simultaneous WD risk factors had received a DB, whereas the respective figures among those without findings were 1.9% and 0.3%.

**Conclusions:**

Self-reported health problems in the HRA, especially multiple simultaneous WD risk factors, predict permanent WD among both genders across occupational groups. Screening WD risk with a self-administered questionnaire is a potential means for identifying high-risk employees for targeting occupational healthcare actions.

## Introduction

The cost of work disability benefits (DB) has become a significant burden to public finances globally (Aumayr-Pintar et al. 2016). Across the OECD countries, public spending on DB is around 2–6% of the gross domestic product (GDP) of the working-age population, depending on the country (OECD 2010). In 2014, about 7% of the Finnish working-age population was on a DB, and the average age of the onset of a permanent DB was 52 (Laaksonen et al. 2016b).

Permanent work disability (WD) is a medico-legal concept (De Boer et al. 2008), which in Finland is defined as having been granted a DB. The benefits programme of the Social Insurance Institution of Finland (Kela) provides coverage for lost income due to medically certified sickness up to 1 year. Thereafter, the DB scheme, operated by pension insurance companies, covers lost income for those eligible. Work ability is assessed on the basis of the employee’s remaining ability to earn an income from work that can reasonably be expected on the basis of their education, previous work history, age, housing conditions, and other social factors. A DB is granted if, based on the attending physician’s statement, the employee’s ability to work is permanently reduced and the expert panel agrees that the decrease in functional capacity and work ability is due to illness or injury. Thus, a granted DB serves a proxy for permanent WD in the present study.

Most Finnish employees use occupational healthcare services (OHS) for all primary healthcare needs. Finnish OHS covers approximately 90% of the total workforce (Lappalainen et al. 2016; Kela-Social Insurance Institution 2019), and carry out preventive and curative health care (Kela-Social Insurance Institution of Finland 2018). One of the primary tasks of OHS in Finland includes protection of employees’ work ability, for which purpose early identification of WD risk would be desirable and, therefore, instruments to tap risks are developed in OHS. Work ability and disability are complex and multifactorial phenomena, determined by personal, socio-demographical, lifestyle- and health-related factors as well as organisational determinants, healthcare management, and legislation. In most countries with disability pension schemes, permanent WD is usually due to a chronic disease (De Boer et al. 2008), which reduces functional capacity and work ability (OECD 2010). The key employee-related predictors of WD reported in observational studies can be divided into demographic factors (e.g., age, gender and educational status) (Laaksonen et al. 2016a; Polvinen et al. 2016; Samuelsson et al. 2012), health status (Karpansalo et al. 2004), and work (e.g., type of occupation) (Haukenes et al. 2011; Borg et al. 2001; Leinonen et al. 2011; Polvinen et al. 2014). Previous studies also suggest that both short-term (Alexanderson et al. 2012; Karlsson et al. 2008; Kivimäki et al. 2004; Virtanen et al. 2006), and long-term (Airaksinen et al. 2018; Gjesdal et al. 2004; Lund et al. 2008) sickness absences (SA) predict new sick leaves and permanent WD.

Some screening questionnaires, such as the World Health Organization’s Health and Work Performance Questionnaire (WHO-HPQ) (Kessler et al. 2003), the Work Ability Index (WAI) (Ilmarinen et al. 1997; Jääskeläinen et al. 2016; Kinnunen and Nätti 2018), and the 12-item Short Form Health Survey (SF-12) (Laaksonen et al. 2011; Roelen et al. 2015), to name a few, are used by researchers, but have not been implemented in broader clinical use. They are laborious to fill out, and more importantly, they are detached from the OHS processes such as occupational health surveillance. Only the WAI has evidence for the capability of predicting permanent WD (Kinnunen and Nätti 2018). Moreover, most of the previous studies have been performed among public sector employees (Airaksinen et al. 2018; Kinnunen and Nätti 2018; Laaksonen et al. 2011), or in specific industries or occupational groups (Kant et al. 2009; Niessen et al. 2012; Roelen et al. 2015; Schouten et al. 2015; Stange et al. 2016). There are different pension act legislations in the public and private sector in Finland, for which reason generalization based on public sector studies to the entire working life should be done with caution. Also, working cultures vary by sector and industry, which is reflected in much higher SA rates in the public sector than in the private sector (Seppänen 2010).

In the present study, we used a health risk appraisal (HRA), which was able to identify blue-collar employees in the construction industry with a high number of SA days in a previous study (Taimela et al. 2007). Especially multimorbidity, i.e., the presence of more than one simultaneous risk factor predicted SA days (Taimela et al. 2007). The HRA presents the results as different risk categories primarily based on self-reported symptoms and health behaviours. The online HRA is widely used in Finland and the Netherlands as a part of preventive occupational health services (OHS) by different providers to recognize employees at risk for SA and to target interventions for those in need. Previous randomised trial also showed that the targeted interventions put in place for employees with high risk of SA, based on the HRA results, were effective in reducing SA days (Taimela et al. 2008a, 2010) and reduced the use of healthcare resources (Taimela et al. 2008b). The predictive ability of the HRA on permanent WD has not been studied before.

We assessed whether the HRA, which is used as an occupational health-care instrument for the prediction of SA, also predicts permanent WD and if so, whether the WD risk increases by the number of self-reported health problems. We hypothesized that the HRA has an independent predictive effect on granted DB as a proxy measure of WD and that the higher the number of risk factors, the higher the WD risk.

## Methods

### Study design, ethics, and setting

The study design was an analysis of prospectively collected register data. We obtained the questionnaire data and the SA data from one OHS provider’s registers. The DB data were obtained from the Finnish Centre for Pensions (ETK), which combines DBs under different pension act legislations into one that is linked to an employee’s career, not to a particular employer, and the coverage of the register is practically 100%. We then combined the data registers using a unique identified, the Finnish social security code. Data privacy was strictly followed.

The Tampere University Research Ethics Board approved the study (ETL code R16074), and it was conducted in accordance with the Declaration of Helsinki.

The study setting was preventive OHS within the framework of the DB legislation in Finland.

### Participants

**Fig. 1:**
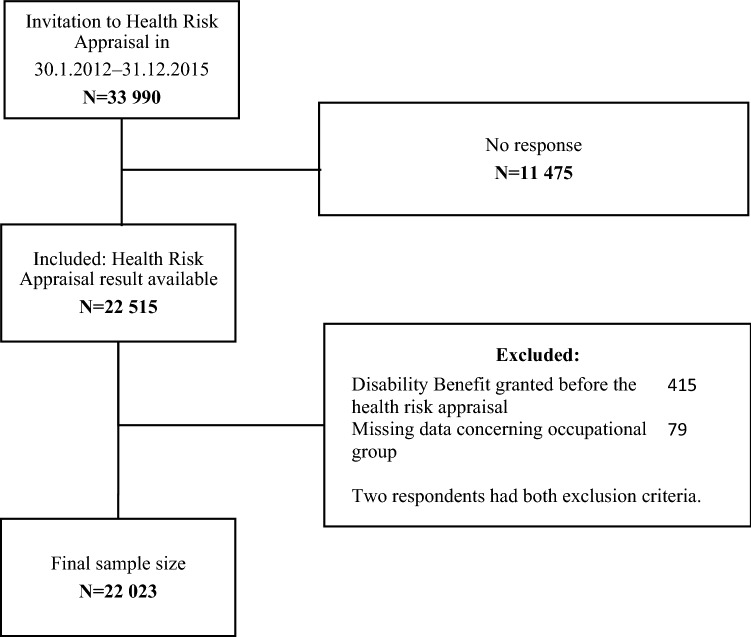
Study flow

The cohort was formed of employees from different companies who acquired their OHS from one nationwide provider, which offers services to a variety of sectors and company sizes. The participants were 19–68 years old Finnish residents, who had completed the HRA (*N* = 22,023) during 2012–2015. We included only the first response. The data on DBs from the national register of ETK covered years 2012–2017. We had access to the complete information on all DB events including their primary and secondary diagnoses based on the International Classification of Diseases, 10th Revision. Figure 1 shows the participants’ exclusion and inclusion criteria.

Inclusion criteria were a completed HRA. An invitation to the HRA had been sent to 33,990 employees during 2012–2015, of which 11,475 had not responded (response rate 66%). We excluded the participants if DB had been previously granted (*N* = 415) or data concerning occupational group were missing (*N* = 79).

### Measurements

#### Explanatory variables

The primary exposure variable of interest in the statistical models was the classified result of the HRA. The result categories in declining priority order are (1) work disability risk, (2) health risk, (3) some symptoms, (4) lifestyle issues, and (5) no findings (Table 1). The first category, labelled as “WD risk”, includes the following self-reported health problems: musculoskeletal problems, depressive symptoms, sleep problems, constant stress and feeling of exhaustion, and doubts about work ability. Within the category “WD risk”, the results were further subdivided by the number of risk factors (one, two, three or more). We combined the “lifestyle issues” and “no findings” categories as the reference class and included the result of the HRA (six categories) as a covariate in the statistical models.

**Table 1:** Criteria for classifying employees into risk appraisal result categories

Topic	Criteria
Work disability risk: at least one of topics below	
Impairment due to musculoskeletal problems at work, OR pain hampers work	Numerical rating scale (0–10) score ≥ 5
	At least moderate pain that affects work ability at least three times a week
Depressive symptoms	Depression score DEPS ≥ 11
Sleep problems	Problems falling asleep or night-time awakenings and daytime sleepiness daily or almost daily
Work-related constant fatigue OR work-related constant stress	Feeling of being squeezed empty
	Feeling tense, strained, nervous and/or anxious because work-related issues are constantly on one’s mind
Doubt regarding work ability	Self-rated future work ability: uncertain of own ability or quite sure of not being able to continue in current job due to health reasons
Health risks: at least one of points below	
Weight problems	Body mass index (BMI) ≥ 30 or ≤ 18.5
Diabetes risk	Diabetes risk score ≥ 11
Excess use of alcohol	Males ≥ 350 ml/week, females ≥ 240 ml/week (expressed as absolute alcohol)
Some symptoms: at least one of points below	
Impairment due to musculoskeletal problems at work	Numerical rating scale (0–10) score = 4
Some depressive symptoms	DEPS score between 8 and 10
Some sleep problems	Problems falling asleep or night-time awakenings and daytime sleepiness 3–5 times a week
A chronic disease	Self-reported chronic diseases diagnosed by doctor
Symptoms	Self-reported symptoms
Lifestyle issues: at least one of points below	
Smoking	Smoking = yes
Physical inactivity	No physical activity during leisure time nor while commuting to work
Overweight	BMI between 25 and 30
No findings	
Previous criteria are not met	

Gender, age, occupational group and the accumulated SA days during the 12 months preceding the HRA were treated as confounding variables. Age was categorized into five classes: ≤ 30, 30–40, 40–50, 50–60, and > 60 years. Occupational group was defined as blue-collar workers, clerical employees, and professionals/managers. The number of SA days 12 months prior to the questionnaire was included as a continuous variable.

#### Work disability

The outcome variable was a granted DB as a proxy measure of permanent WD, and it was operationalized dichotomously as a granted DB: yes/no. The mean follow-up time was 3.5 years (SD 1.1, range from 3 days to 5.9 years, median 3.3 years) from the date of the survey response.

DBs in our study consist of four categories as follows: (1) full and (2) partial disability pension, or (3) full and (4) partial rehabilitation subsidy. A DB is granted if the remaining maximum capacity to work is 40% (2/5), as in the case of a full-time benefit; or 60% (3/5), as in the case of a partial benefit. The duration of the DB can be until further notice or for a temporary period. The common requirement in all categories of DB is the permanent nature of reduction of work ability.

### Statistical methods

It has been suggested that gender should not be treated as a covariate and that the analyses should be carried out separately by gender (Messing et al. 2003). Indeed, there were complex interactions in our study between gender and occupational groups (data not shown), and we performed all analyses stratified by gender.

We present descriptive statistics to describe the eligibility categories and the most common health issues that lead to DBs. We compared the demographic characteristics of the participants and non-participants using *t* test and Chi-squared test. We used the cumulative incidence function (CIF) to illustrate the difference between the HRA risk categories (Kim 2007), and the Fine–Gray proportional hazards model to estimate how HRA categories, age and occupational group affected the probability of events, i.e. a granted DB, prior to a follow-up (Fine and Gray 1999). The Fine–Gray model provides hazard ratio (HR) estimates to describe the relative effect of covariates, which are then also associated with the probability of a DB occurring over time. Model 1 included only the HRA categories; and Model 2, the fully adjusted model, also included age, occupational group and earlier SA.

The statistical analyses were performed using the cmprsk library and R 3.4.4 software.

## Results

The mean age of the participants was 45.5 years (SD 11.1; range 19.1–68.0), 59% (*N* = 12933) were female, 31% (*N* = 6807) were blue-collar workers, 55% (*N* = 12072) were clerical employees, and 14% (*N* = 3144) belonged to the professional or manager category. The non-respondents were slightly younger (average age 44.2 years, SD 12.3; *t* = − 9.0; *p* < 0.0001) than the respondents on the average. Also, males were less likely to respond than females with response rates 60% and 71%, respectively (Chi square 425.5; *p* < 0.0001). The response rates were almost identical among blue-collar workers (65%), clerical employees (67%) and experts/managers (66%), (Chi square 14.3; *p* = 0.0007).

A total of 379 participants in the cohort were granted a DB on the average 2 years (range from 3 days to 5.7 years) after the HRA. The overall annual incidence of a DB was 0.29%: 0.33% among the females and 0.23% among the males (*p* = 0.23). In the Fine–Gray model, which included gender as the explanatory variable and age, occupational group, and SA days before questionnaire as confounders, the HR for gender was 1.2 (0.9–1.5; males as the reference).

Of those who had received a DB, 149 (39%) participants had a primary diagnosis of a musculoskeletal disorder and 80 (21%) participants had a primary diagnosis of a mental or behavioural disorder (Table 2). Fifteen participants had both musculoskeletal and mental or behavioural diagnoses simultaneously (4% of all DBs).

**Table 2:** Distribution of causes of disability benefits according to the ICD-10 classification (International Classification of Diseases, 10th revision)

Cause of disability benefit according to ICD-10 classification			The first diagnosis		The second diagnosis	
			*N*	%	*N*	%
M Diseases of musculoskeletal system and connective tissue			**149**	**39**	**120**	**32**
M40–M54	Spinal disorders		70	18	57	15
M17	Osteoarthritis of the knee		19	5	9	2
M75	Shoulder disorders		13	3	20	5
M19	Other osteoarthritis		10	3	8	2
	Other musculoskeletal disorders		37	10	26	7
F Mental and behavioural disorders			**80**	**21**	**40**	**11**
F32–F34	Mood (affective disorders)		56	15	8	2
F31	Bipolar affective disorder		9	2	1	< 1
F20–F29	Schizophrenia, schizotypal and delusional disorders		7	2	0	0
	Other mental and behavioural disorders		8	2	31	8
C Neoplasms			**40**	**11**	**11**	**3**
C50	Malignant neoplasm of the breast		13	3	2	< 1
C15–C26	Malignant neoplasm of digestive organs		9	2	0	0
C51–68	Malignant neoplasm of genital organs and urinary tract Other malignant neoplasms		5	1	2	< 1
			13	3	7	2
G Diseases of the nervous system			**35**	**9**	**15**	**4**
G35	Multiple sclerosis		6	2	0	0
G20	Parkinson’s disease		4	1	0	0
G24	Dystonia Other diseases of the nervous system		3	< 1	0	0
			22	6	15	4
I Diseases of the circulatory system			**22**	**6**	**17**	**4**
I60–I69	Cerebrovascular diseases		12	3	1	< 1
I20–I25	Ischaemic heart disease		2	< 1	10	3
I42	Cardiomyopathy		3	< 1	0	0
I48	Atrial fibrillation and flutter Other diseases of the circulatory system		2	< 1	2	< 1
			3	< 1	5	1
S Injuries			**15**	**4**	**9**	**2**
S40–S69	Injuries to upper limbs		8	2	3	< 1
S00–S19	Injuries to head and neck		3	< 1	1	< 1
S70–S99	Injuries to lower limbs		4	1	5	1
Others			**38**	**10**	**32**	**8**
H00–H59	Diseases of the eyes and adnexa		6	2	5	1
Q00–Q99	Congenital malformations		5	1	0	0
H81–H93	Diseases of the ears and mastoid process		4	1	3	< 1
T90–T93	Sequelae of injuries		4	1	0	0
Z59	Problems related to housing and economic		4	1	0	0
N08–N30	Diseases of the genitourinary system		3	< 1	0	0
E10–E11	Diabetes mellitus		2	< 1	3	< 1
E66	Obesity		1	< 1	5	1
Miscellaneous (*N* per category < 3)			9	2	16	4
No information or second reason not registered			**0**	**0**	**135**	**36**

Bold values denote the sum of conditions per category

A pension application may include multiple diagnoses, i.e., several ICD-10 classes

**Fig. 2:**
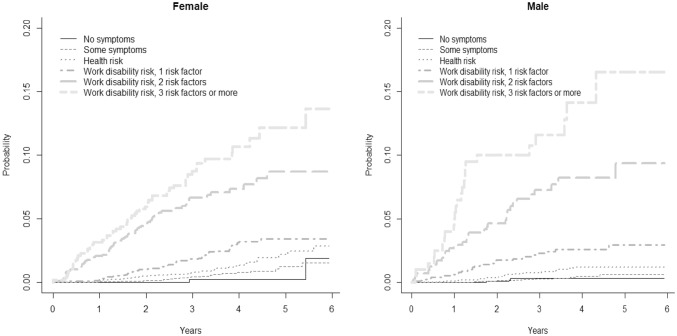
Cumulative incidence of disability benefits over 6-year follow-up period by different health risk appraisal risk groups among females and males

Figure 2 presents the cumulative incidence of the DBs during the 6-year follow-up period in the HRA categories. The HRA “work disability risk” category predicted DB and there was a dose–response relationship between the number of WD risk factors and the probability of ending up on DB. Of the females with three or more WD risk factors, 14% received a DB at 6 years, while the respective figure among the males was 17%. The respective figures for those in the HRA “no symptoms” category was 1.9% for females and 0.3% for males.

In the fully adjusted Fine–Gray model, the HRA WD risk categories, age, occupational group, and SA before the HRA questionnaire predicted the probability of DB for both genders in an additive manner (Table 3). In the unadjusted model (Model 1), the HR for the probability of a DB was 36.2 (8.8–148.4) for the females and 47.7 (14.4–158.1) for the males in the HRA WD risk groups with three and more risk factors. When all covariates were included (Model 2), HR decreased among both genders, and was 17.3 (4.2–71.7) for the females, and 18.2 (5.4–60.8) for the males (Table 3). The same was also seen in the HRA WD risk categories with one and two risk factors. In the fully adjusted model, HR by age was the highest in the 50- to 60-year age group, among both genders [12.9 (4.8–35.2) for females, and 26.4 (3.6–192.8) for males]. By occupational group, blue-collar workers had the highest HRs [3.6 (1.7–7.9) for females and 2.4 (1.2–4.9) for males]. The higher the number of SA days prior to the survey, the higher the HR among both genders.

**Table 3:** Probability of disability benefit by covariates over time

Females	*N*	Model 1		Model 2	
Explanatory variable		HR	95% CI	HR	95% CI
No findings or lifestyle issues only	711	1.00	Ref.	1.00	Ref.
Some symptoms	5101	2.27	[0.55–9.43]	1.95	[0.47–8.09]
Health risk	3021	**4.33**	**[1.05–17.93]**	3.06	[0.74–12.64]
Work disability risk, 1 risk factor	2560	**8.48**	**[2.08–34.63]**	**5.95**	**[1.46–24.32]**
Work disability risk, 2 risk factors	969	**25.87**	**[6.35–105.44]**	**14.98**	**[3.68–61.00]**
Work disability risk, 3 and more risk factors	571	**36.23**	**[8.85–148.37]**	**17.34**	**[4.19–71.75]**
Age ≤ 30	1343			1.00	Ref.
Age > 30 and ≤ 40	2750			2.46	[0.83–7.25]
Age > 40 and ≤ 50	3578			**3.94**	**[1.39–11.09]**
Age > 50 and ≤ 60	4102			**12.92**	**[4.75–35.16]**
Age > 60	1160			**3.76**	**[1.24–11.41]**
Occupational group: professional and manager	1456			1.00	Ref.
Occupational group: clerical	7452			**2.53**	**[1.18–5.44]**
Occupational group: blue-collar	4025			**3.64**	**[1.68–7.89]**
Sick leave days before questionnaire				**1.009**	**[1.006–1.012]**

Males	*N*	Model 1		Model 2	
Explanatory variable		HR	95% CI	HR	95% CI
No findings or lifestyle issues only	1040	1.00	Ref.	1.00	Ref.
Some symptoms	3702	1.22	[0.35–4.27]	0.89	[0.25–3.18]
Health risk	2478	3.19	[0.96–10.64]	1.96	[0.59–6.50]
Work disability risk, 1 risk factor	1260	**8.22**	**[2.51–26.89]**	**3.84**	**[1.17–12.57]**
Work disability risk, 2 risk factors	410	**26.79**	**[8.20–87.52]**	**10.86**	**[3.34–35.29]**
Work disability risk, 3 and more risk factors	200	**47.75**	**[14.42–158.11]**	**18.17**	**[5.43–60.81]**
Age ≤ 30	947			1.00	Ref.
Age > 30 and ≤ 40	2310			2.19	[0.25–18.94]
Age > 40 and ≤ 50	2426			6.62	[0.87–50.14]
Age > 50 and ≤ 60	2554			**26.43**	**[3.62–192.75]**
Age > 60	853			**11.93**	**[1.53–93.17]**
Occupational group: professional and manager	1688			1.00	Ref.
Occupational group: clerical	4620			1.36	[0.68–2.72]
Occupational group: blue-collar	2782			**2.41**	**[1.19–4.90]**
Sick leave days before questionnaire				**1.011**	**[1.008–1.014]**

Bold values denote statistical significance at the p < 0.05 level

Subdistribution hazard ratios obtained from the Fine–Gray model describe the relative effect of covariates on the subdistribution hazard function. Covariates in this model can be interpreted as having an effect on the cumulative incidence function of disability benefits occurring over follow-up. Model 1 includes the health risk appraisal risk classes only. Fully adjusted Model 2 includes age, occupational group and prior sick leave days as covariates

## Discussion

Self-reported health problems in the HRA—musculoskeletal problems, depressive symptoms, sleep problems, constant stress and feeling of exhaustion, and doubts about work ability—predicted WD in both genders, in all occupational groups. Of note, the larger the number of these problems, labelled as “WD risk factors”, the higher was the risk for WD. In Finland, the two largest categories of the causes of permanent WD are musculoskeletal disorders and mental and behavioural disorders (Social Insurance Institution of Finland 2019; Official Statistics 2018). Also, problems with sleep (Haaramo et al. 2012), constant stress (Juvani et al. 2018), exhaustion (Ahola et al. 2009), and attitudes towards work ability (Kinnunen and Nätti 2018) have predicted SA and/or WD in earlier studies. It seems that using a questionnaire for self-rating of relevant symptoms is a valid way to identify individuals at risk of WD, as the HRs were relatively high in our study. Age, occupational group and earlier SA also predicted future DB in an additive manner. By age, the risk of DB was the highest in the 50- to 60-year age group, among both genders.

Reporting health problems in the HRA had a strong, independent predictive value for future DB. Earlier studies have provided evidence that self-reports in a questionnaire predict DB (Bethge et al. 2017). The Work Ability Index (WAI) (Ilmarinen 2009) has been used in countries such as Finland and other Scandinavian countries, the Netherlands, and Germany (Bethge et al. 2017; Jääskeläinen et al. 2016). Two longitudinal studies have reported that the risk of a granted DB was higher among employees with poorer WAI scores [HR 7.8; 95% CI 2.6–23.4 (Bethge et al., 2017), and HR 5.0; 95% CI 4.4–5.6 (Jääskeläinen et al. 2016)]. Our results provide further support for earlier studies that perceived health and symptoms predict WD. Of note, the HRs in our study were exceptionally high among those reporting multiple “WD risk factors”, i.e., health problems. Age has been a predictor of a future DB in previous studies (Gjesdal et al. 2004; Karlsson et al. 2008). By age, the risk of WD was the highest in the 50- to 60-year age group in our study population. This might be because of a “healthy worker survivor effect” (Osmotherly and Attia 2006), which means that only the healthiest and strongest remain in working life, and those who became unfit during their employment tend to leave working life earlier (Osmotherly and Attia 2006). This effect was notable in our study, in which the over-60 age group had a lower rate of DBs than the 50- to 60-year age group. The HRs for DBs were highest among the blue-collar employees in the present study. This is in line with a previous study, in which the data were drawn from seven independent studies in Finland, France, the UK and the USA, and which reported an association with a low occupational grade and increased risks of health-related exit from work (Carr et al. 2018). A Finnish cohort study found that higher occupational classes are two times more likely to continue working beyond retirement age than lower occupational classes, while another cohort study found that hospitalization was slightly more associated with increased DB in the lower occupational classes. These studies indicate that lower occupational classes have poorer health. In our study, the gender difference was not statistically significant in terms of the annual incidence of granted DB. The findings of previous studies in this respect are contradictory. A previous prospective study found no overall gender difference in DB rates (Gjesdal et al. 2004), whereas other studies have found gender differences. A Finnish register-based retrospective study found a gender difference between different SA trajectories, which led to DB (Laaksonen et al. 2016a), although the associations with socio-demographic variables were weak. A Swedish twin cohort found that females are at a higher risk of DB (HR 1.31; 1.26–1.37) than males (HR 1.00; reference). In the present study, we found that earlier SA days predicted future DB, which is in line with previous studies (Kivimäki et al. 2004; Laaksonen et al. 2016a; Lund et al. 2008; Øyeflaten et al. 2014; Salonen et al. 2018).

The key strength of our study is its prospectively collected, extensive, registry-based data from various industries. We were also able to control potential confounders such as age, gender and occupational group. The archival data of DBs at the ETK were comprehensive and virtually no data were lost to follow-up (Finnish Centre for Pensions 2018). We combined all four DB categories as one as the proxy measure for WD: this way, no data were lost and virtually all the DB recipients had had at least 1 year of sickness allowance before the granted DB. Another strength is that we used the HRA, which can identify employees with a high number of SA days (Taimela et al. 2007). The follow-up continued at least 2 years after the completed HRA. Sickness allowance is paid for a maximum of 1 year after the onset of WD in Finland and the DB decision is typically made shortly after the sickness allowance period. Thus, the 2-year follow-up period was long enough to detect all new potential DB receivers.

We chose to use the Fine–Gray model to estimate the effect of the covariates on the rate at which WD occurs. Although the model was perhaps not able to deal with all the complexity associated with our data, among computationally feasible approaches, it is more appropriate than, e.g., the Kaplan–Meier survival analysis that tends to overestimate cumulative incidence of health-related events (Lacny et al. 2018). Besides, it was easier to add variables to Fine–Gray model than for example in Kaplan–Meier. Moreover, we prefer talking about cumulative hazards to “survival at work” conceptually. However, interpretation of the HR estimates from the Fine–Gray model is not straightforward. We recommend interpreting the covariates as having an effect on the incidence of WD (i.e., on the CIF). However, the magnitude of the relative effect of the covariate on the subdistribution hazard function is different from the magnitude of the effect of the covariate on the CIF. Yet one can conclude that if a variable increases the subdistribution hazard function, it will also increase the incidence of the event. However, one cannot infer that the exact magnitudes of these two effects are the same (Austin and Fine 2017).

We did not have access to the statutory accident insurance data, so WD resulting from accidents at work, occupational diseases, and traffic accidents are not included in our study. Moreover, our results can only cautiously be generalized to the entire working-age population, because people outside working life were not involved in our study. Another limitation of study is the potential selection bias due to differences between respondents and non-respondents. “Healthy worker effect” might be present if employees with worse health level had not responded or they are less likely to hire (Chowdhury et al. 2017). Similar bias would potentially result from a “healthy worker survival effect”, which means that only healthiest and strongest will remain in the working life (Nordström et al. 2016). All this might underestimate the associations. It may also be possible that the healthiest employees might not respond to the HRA, which would have an opposite effect on our estimates.

Some DB criteria are comparable between countries, such as requirements for a health condition in relation to work and the permanence of the condition (De Boer et al. 2008). However, the implementation of the legislation varies between countries (OECD 2010) and, therefore, our results must be interpreted with caution in the international context. However, we assume that the phenomenon itself—severe self-rated health problems predict WD—manifests in different medico-legal contexts.

The outcome of interest was rare in the entire population in our study, which is visible in the wide confidence limits for the different risk categories for both genders. However, permanent WD is very costly for society (OECD 2010), and the underlying diseases and disorders are a burden to disabled individuals in addition to their lost income. Hence, it is important to identify predictors of SA and WD and to determine how to prevent WD. Practical tools are needed to identify the risk factors for WD and to target interventions for those in need. The HRA used in the present study seems to function in OHS as a practical tool to recognize employees at increased risk for SA and DB early for the purpose of targeting OHS actions to those who need special support in maintaining their work ability. The aggregated results may also be utilised in promoting sustainable working conditions.

Our results indicate high HRs for permanent WD among employees belonging to in the HRA work disability risk category and provide further support that in addition to prior absence from work, physically demanding work and age, self-reported health problems play an independent role in identifying employees who are at an increased risk of WD. Further research is needed to assess the effectiveness and cost-effectiveness of targeted health surveillance among the risk groups.

## Data Availability

No additional data are available due to data privacy reasons.
